# Effect of construction defects on construction and demolition waste management in building construction: a systematic literature review

**DOI:** 10.1093/inteam/vjae026

**Published:** 2025-01-06

**Authors:** Gayani Karunasena, Argaw Gurmu, Salman Shooshtarian, Nilupa Udawatta, C Savindi Ranthika Perera, Tayyab Maqsood

**Affiliations:** School of Architecture and Built Environment, Deakin University, Geelong, VIC, Australia; School of Architecture and Built Environment, Deakin University, Geelong, VIC, Australia; School of Property, Construction and Project Management, RMIT University, Melbourne, VIC, Australia; School of Architecture and Built Environment, Deakin University, Geelong, VIC, Australia; Department of Architecture, Monash University, Melbourne, VIC, Australia; School of Property, Construction and Project Management, RMIT University, Melbourne, VIC, Australia

**Keywords:** building projects, building defects, construction rework, construction waste, waste minimization

## Abstract

The occurrence of defects in building construction projects is a significant issue, leading to increased construction waste and negatively affecting sustainability and overall project performance. Despite its critical nature, the specific relationship between construction defects and waste generation has been underexplored in the literature. This study seeks to address this gap by conducting a systematic literature review of relevant publications. The research followed the Preferred Reporting Items for Systematic Reviews and Meta-Analyses (PRISMA) guidelines, conducting an extensive search across databases like Scopus and Web of Science, which resulted in the identification and content analysis of 59 pertinent articles. The findings reveal that poor workmanship, inadequate planning and scheduling, and frequent design changes are the primary causes of defect-related waste. Additionally, the study identified 12 themes, noting that the quantification of the cost of quality and the association between defect, rework and waste have not been thoroughly analyzed. The study's implications are twofold: Theoretically, it contributes to the academic understanding of the link between construction defects and waste generation, laying a foundation for future research in this area. Practically, it underscores the need for improved industry practices, such as enhanced training for construction workers, more rigorous project planning, and stricter adherence to design and specifications, to mitigate defect-related waste and promote sustainable construction practices.

## Teaser

**Key points**
 

- The study reveals that poor workmanship, inadequate planning and scheduling, and frequent design changes are the primary causes of defect-related waste.
- The study proposes industrial practices such as enhanced training for construction workers, more rigorous project planning, and stricter adherence to design and specifications to mitigate defect-related waste.
- The study also identifies the growing financial implications of construction defects, particularly in the context of rising waste disposal costs and increased complexities in building insurance claims.
- The study reveals the link between construction defects and waste generation, laying a foundation for future research.

## Introduction

The construction industry faces significant challenges related to construction and demolition (C&D) waste generation and building defects. Although both issues have been extensively studied, they are often treated as separate areas of inquiry. The literature on C&D waste typically focuses on sources, management strategies, and environmental impacts (Shooshtarian et al., 2024; Sweis et al., 2021; Zheng et al., 2017), whereas research on building defects examines causes, prevention, and consequences in terms of structural integrity, safety, and cost implications (Ahzahar et al., 2011; Gurmu et al., 2023a; Pamera & Gurmu, 2020). Despite the considerable body of research in both domains, the relationship between building defects and C&D waste generation has not been explicitly explored, revealing a critical gap in understanding.

Construction defects are errors or imperfections that deviate from specified requirements and can occur at various stages of a project, from design and planning to construction and occupancy (Macarulla et al., 2013). These defects manifest in multiple forms, including structural flaws, functional deficiencies, aesthetic issues, and safety hazards (Ahzahar et al., 2011). They are generally categorized into structural defects, resulting from inadequate design, materials, or workmanship, and nonstructural defects, often arising from poor workmanship or defective materials (Ali, 2019; Gurmu et al., 2023b; Shooshtarian et al., 2023a). Construction defects have significant consequences, such as increased construction costs (Sandanayake et al., 2021), reputational damage to construction companies (Gurmu et al., 2021), and distress for building occupants. Crucially, these defects also contribute to C&D waste, as defective components often require demolition, thereby increasing waste volume.

The construction industry has long been criticized for its extensive waste generation, with approximately 35% of construction waste ending up in landfills worldwide (Ajayi et al., 2015; Sweis et al., 2021; Zheng et al., 2017). This waste poses severe environmental, social, and economic challenges. Environmentally, C&D waste contributes to global warming through methane emissions from landfills and air and water pollution (Zhang et al., 2019). Socially, landfill sites degrade the quality of life for nearby communities due to health risks and visual pollution (Chinda et al., 2012). Economically, improper C&D waste management can lead to increased project costs and delays, with material waste adding between 11% and 30% to construction project expenses in regions such as the U.K., Netherlands, and Hong Kong (John & Itodo, 2013; Nagapan et al., 2012). Given that construction defects often contribute to C&D waste, understanding their role is crucial for addressing these broader challenges. Although defects and waste have been studied separately, their intersection remains underexplored.

Although construction defects are a known contributor to waste generation and have been a prominent topic for over two decades (Gurmu et al., 2022; Johnston & Reid, 2019), the link between these defects and C&D waste has received little attention. Anecdotal evidence suggests that defective building components, often demolished due to poor workmanship or noncompliance with standards, contribute significantly to C&D waste. However, no comprehensive studies have been conducted to explore this relationship in depth. Although waste and defects have been individually studied, there is a lack of research examining how these two issues intersect.

This study aims to address this gap by conducting a thorough systematic review of the literature to explore and clarify the relationship between building defects and C&D waste generation. By synthesizing existing knowledge, identifying patterns and trends, and highlighting areas for further research, this review seeks to contribute to more integrated approaches for managing both construction quality and waste. Ultimately, this research will inform better construction practices, waste management strategies, and policy development aimed at minimizing waste generation and enhancing the sustainability of construction projects. In this research context, defect-related waste refers to materials, labor, time, and resources wasted due to errors, flaws, or deficiencies in construction processes, components, or workmanship.

## Research methodology

This research used a qualitative approach to address the research aim. The following sections describe the details of data collection and analysis procedures.

### Data collection

**Figure 1.**
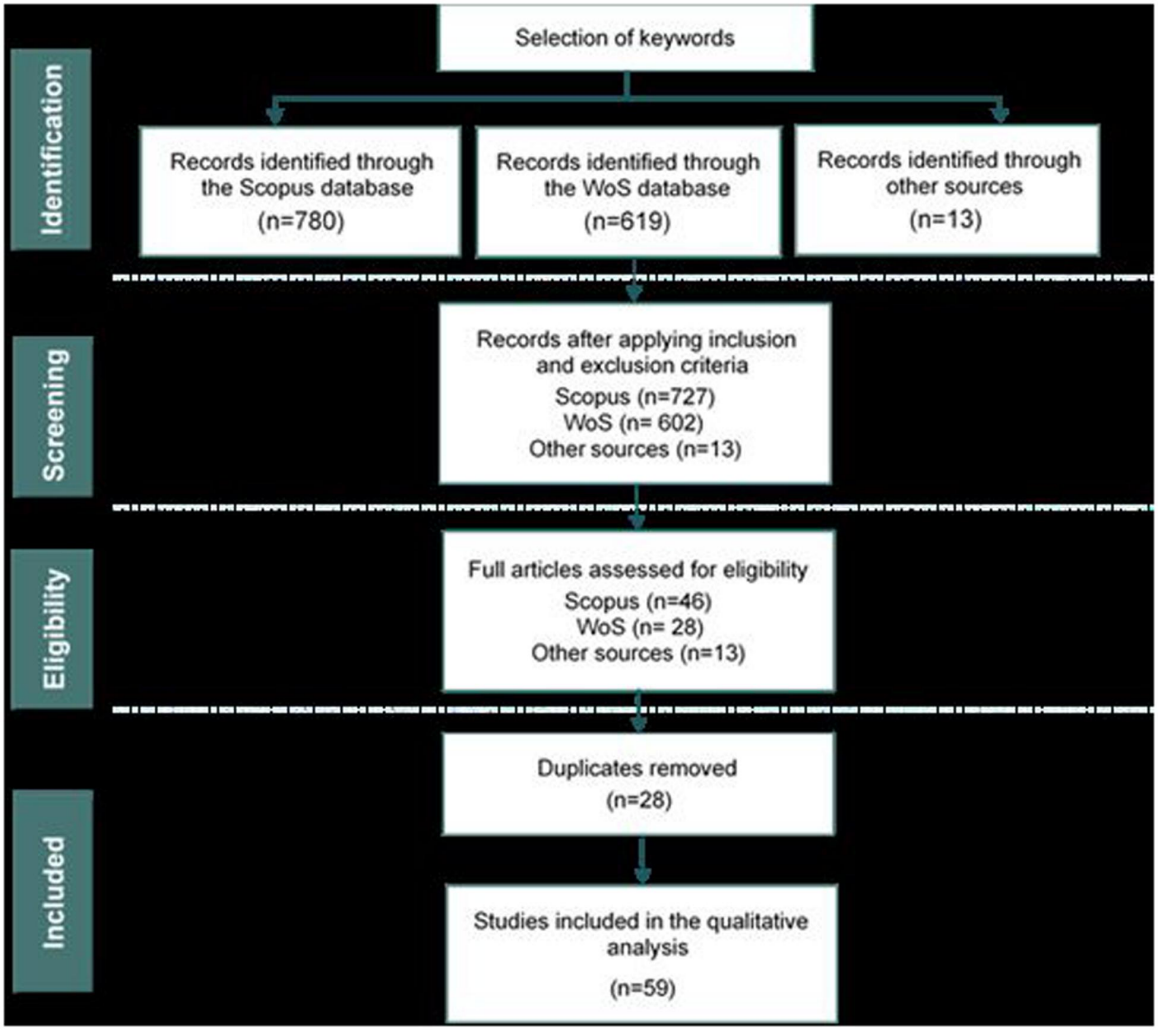
Research flowchart (Preferred Reporting Items for Systematic Reviews and Meta-Analyses [PRISMA]). Source: adapted from Moher et al. (2009).

Qualitative secondary data were collected using a systematic literature review in August 2023. The literature review process was guided by Preferred Reporting Items for Systematic Reviews and Meta-Analyses (PRISMA) guidelines (Moher et al., 2009). According to Shahruddin and Zairul (2020), the PRISMA method enables researchers to perform their analysis of scientific and academic literature from extensive databases by (1) using keyword and search strategies to identify substantial databases containing relevant materials, (2) applying inclusion and exclusion criteria to screen and select suitable studies, and (3) conducting an eligibility process to appraise the pertinent literature and extract data for analysis. Figure 1 outlines the steps involved in the PRISMA model adopted in this study.

The data in this study were primarily collected from two academic databases: Scopus and Web of Science (WoS). This choice was informed by the fact that these databases are widely used by scholars and offer comprehensive resources (Chadegani et al., 2013; Mongeon & Paul-Hus, 2016). The initial search was conducted using the “title/abstract/keyword” field in Scopus and the “all fields” option in WoS, covering publications up to August 2023. The search strings used Boolean operators and keywords such as (“defect*” OR “rework”) AND “waste”) AND (“residential” OR “construction” OR “building”) to identify relevant sources.

The selection of literature was guided by specific inclusion and exclusion criteria to ensure relevance and quality. Studies published up to August 2023 were included to capture the most recent trends and findings. Only English-language publications were considered, as they are the most accessible and interpretable by the research team. The review focused on journal articles, conference proceedings, book series, and books, as these sources provide comprehensive and peer-reviewed information. Studies addressing defects, rework, and waste within residential, construction, or building contexts were selected, with relevance determined through keyword searches and abstract reviews.

Conversely, studies published in languages other than English were excluded due to potential translation biases and the research team's linguistic limitations. Nonacademic publications, such as industry reports, magazine articles, and blogs, were also excluded to maintain academic rigor. Additionally, publications not focusing on the intersection of defects, rework, and waste in the relevant contexts were excluded after a thorough abstract review.

To further expand the search, a snowballing technique was used, leading to the inclusion of 13 strongly related publications extracted from Google Scholar (Jalali & Wohlin, 2012). The initial keyword search yielded 727 publications in Scopus and 602 in WoS. To assess the publications for eligibility, the abstracts of each publication were reviewed to assess their relevance to the current research, resulting in a final selection of 59 publications after removing duplicates (28 publications) for detailed analysis. The bibliographic information for these final publications was collected from Scopus and WoS, forming the dataset for this study.

### Data analysis

This study used a combination of thematic and descriptive analyses to comprehensively examine the literature on construction defects and their impact on C&D waste. Thematic analysis was the primary method used, which is particularly effective in synthesizing a wide range of literature by identifying and organizing recurring themes and patterns. This approach not only allows for a deeper understanding of the subject matter but also facilitates the identification of key issues, trends, and gaps within the existing body of research. By systematically analyzing the themes emerging from the literature, the study was able to offer nuanced insights into the complex relationship between construction defects and waste generation.

In addition to thematic analysis, descriptive analyses were used as a secondary method to map out the broader research landscape. Descriptive analyses are instrumental in providing a comprehensive overview of the existing studies, enabling the identification of trends, frequency distributions, and other statistical patterns in the literature. This method is particularly valuable for summarizing the scope and focus of research in a given field, highlighting areas that have received significant attention as well as those that remain underexplored.

The integration of thematic and descriptive analyses in this study provided a robust methodological framework that not only allowed for a detailed exploration of the existing research but also supported the identification of emerging trends and gaps that warrant further investigation. This approach has been widely adopted in review studies across various fields, including construction defect management (Gurmu et al., 2022), C&D waste management (Chen et al., 2018), and circular economy (Camón Luis & Celma, 2020). By leveraging these analytical methods, the study contributes to a deeper understanding of the current research landscape and offers valuable insights that can inform future studies and industry practices in construction defect and waste management.

## Findings

### Descriptive analysis

**Figure 2.**
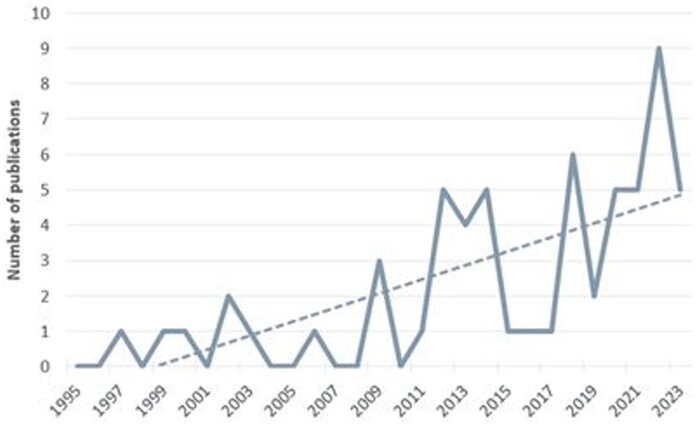
Publication frequency by year.

Over the past three decades, there has been a noticeable increase in research focused on studying defects and waste within the construction sector (Figure 2). Prior to 2006, these areas received limited attention, but a significant rise in research activity began in 2009. By 2022, the number of publications had reached nine, indicating a growing interest in understanding the relationship between construction defects and waste. This upward trend aligns with a broader recognition of the significant impact that defects have on waste generation.

**Figure 3.**
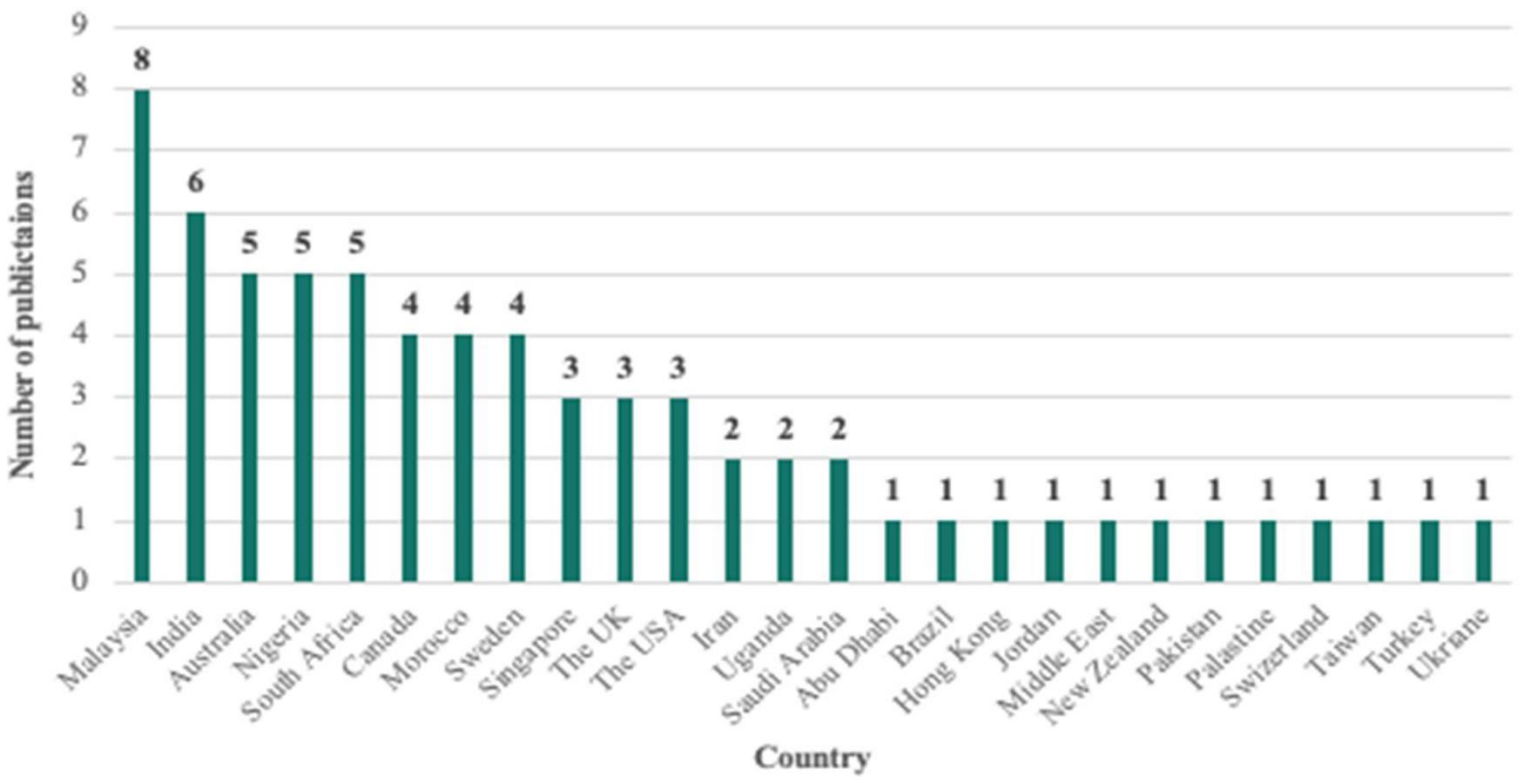
Distribution of publications by country.

The analysis reveals that 79% (42) of the studies were published in academic journals, with the remaining works spread across books (2) and conference papers (15), highlighting the academic community's increasing focus on this topic. These publications span 26 different countries, with Malaysia and India leading in contributions. This geographical diversity underscores the global relevance of the issue, particularly in regions where construction activities are rapidly expanding. Malaysia's prominence as the top contributor to this body of literature could be attributed to its rapid urbanization and economic growth, which have driven extensive construction activities in recent decades. Additionally, Malaysia has implemented comprehensive regulatory frameworks and policies aimed at sustainable construction and waste management. These factors likely contribute to Malaysia's leadership in research output on construction defects and C&D waste. In India, for instance, the introduction of the Construction & Demolition Waste Management Rules (2016) might have spurred significant research, reflecting a growing recognition of the link between construction practices and waste management. Understanding this global research landscape provides a broader context for the study's aim, emphasizing the need for more focused research on the intersection of defects and C&D waste, especially in regions with high construction activity and evolving regulations (Figure 3).

### Results of thematic analysis

**Figure 4.**
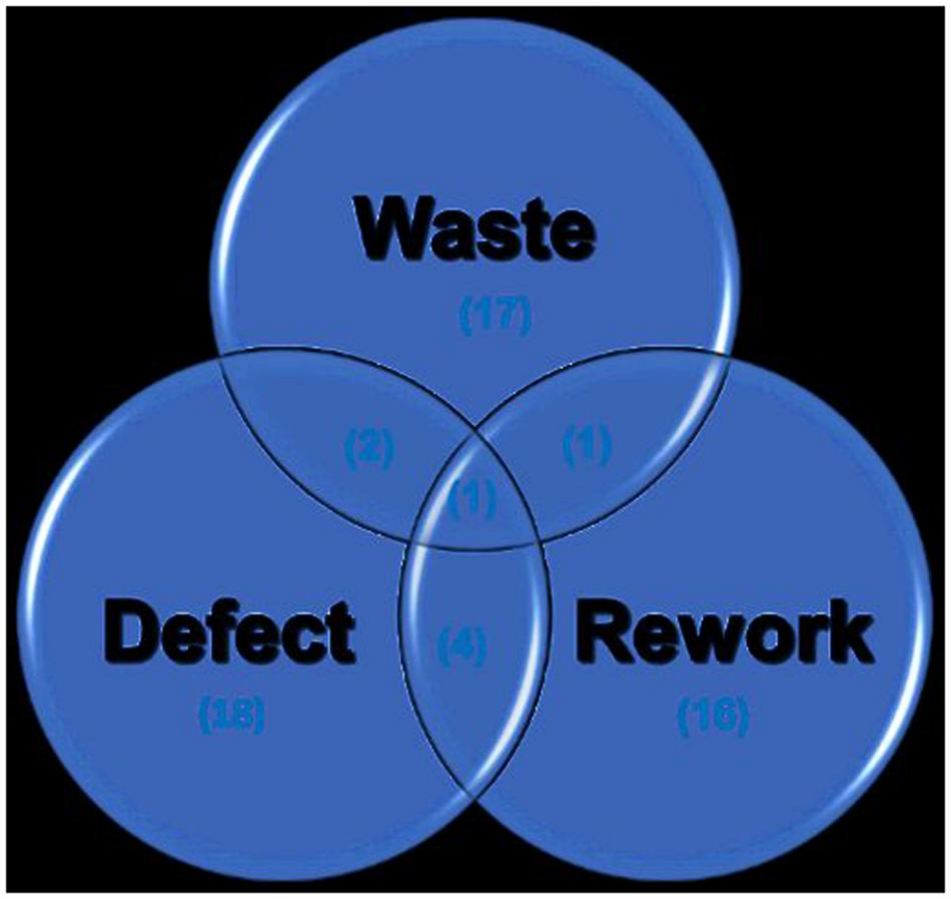
The major themes of research in the reviewed studies and the frequencies of resources.

The thematic analysis identified three primary research themes: waste, defects, and rework. However, only a limited number of studies (approximately 17%) have explored the intersection of defects and waste (Figure 4). Most research has focused on these themes individually rather than their interactions. Notably, only two studies addressed both defects and waste (Dahlgaard & Dahlgaard, 2002; Ko & Li, 2014), and one study explored the relationship between rework and waste (Desai et al., 2023). This highlights a significant gap in understanding how defects specifically contribute to C&D waste.

Among the few studies that explored this intersection, Mahamid (2022) used a survey to capture the views of construction experts on the causes of waste and rework. The findings highlighted “untrained laborers” and “redoing work due to poor workmanship” as major contributors to both waste generation and rework. Similarly, Desai et al., (2023) examined the application of artificial intelligence (AI) and building information modeling (BIM) to minimize defects, rework, and waste, representing one of the rare attempts to study all three aspects together.

**Figure 5.**
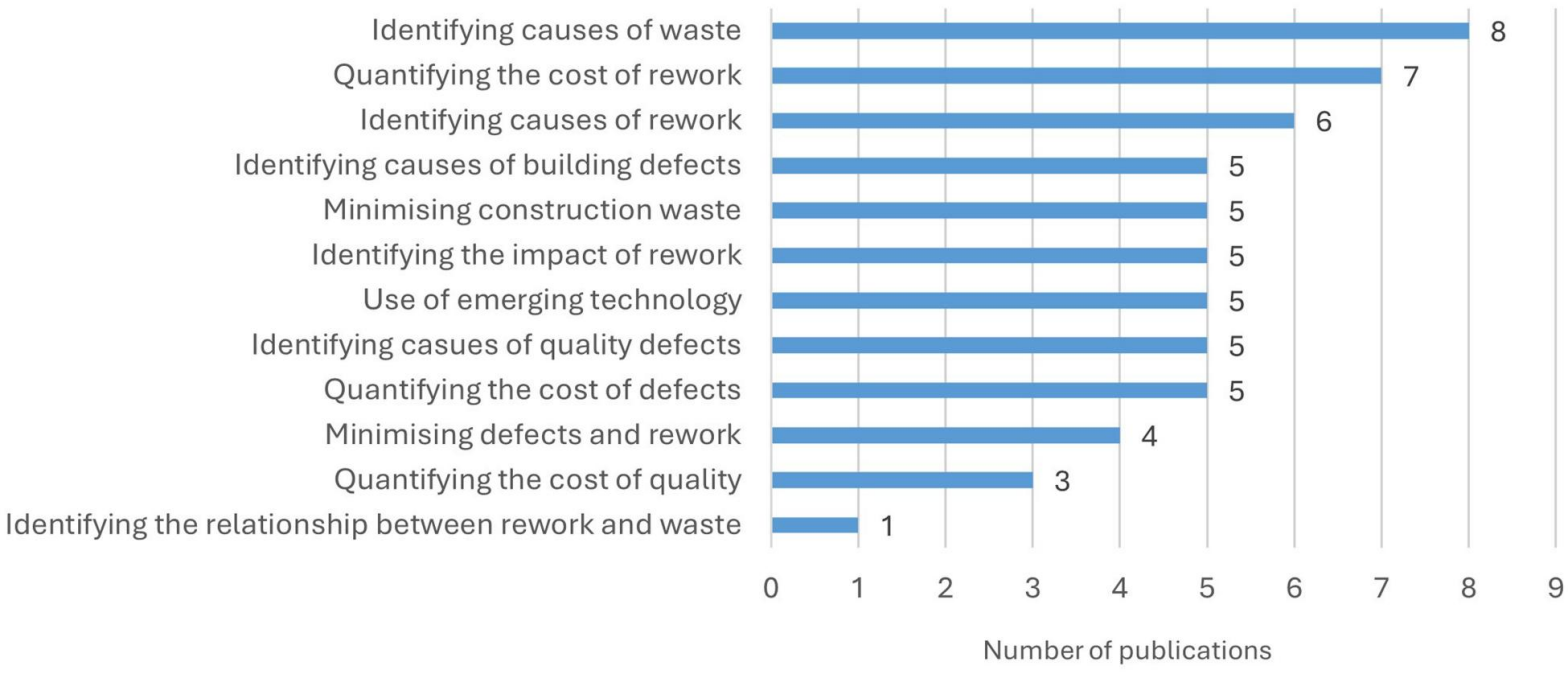
Detailed area of the research based on the subthemes.

To better understand the scope of the studies reviewed, further analyses were conducted. As shown in Figure 5, 12 subthemes have emerged where “identifying waste causes,” “rework causes,” and “quantifying the cost rework” are the key subthemes. The following sections scrutinize these subthemes in detail.

### Causes of waste

The literature analysis revealed a range of causes for waste generation in construction sites (Table 1). For each study reviewed, only the main five causes are extracted and presented in Table 1. The results show that the major causes are poor workmanship, inadequate planning and scheduling, and design changes. Poor workmanship relates to the use of untrained laborers (Mahamid, 2022) on construction sites, mistakes made by laborers during construction (Nagapan et al., 2012), poor material handling (Musarat et al., 2023), and the lack of skills and experience among workers (Fitchett & Rambuwani, 2022). These deficiencies in workmanship often lead to construction defects, which are particularly critical as they result directly in rework and repairs, both of which significantly contribute to C&D waste. For instance, mistakes made by untrained laborers often necessitate remedial work, involving the demolition of faulty building elements and the disposal of construction materials. This cycle of rework not only wastes resources but also amplifies the volume of waste generated on-site. Furthermore, defects such as improper material handling or installation errors can lead to the rejection or replacement of materials, further exacerbating waste production.

**Table 1.** Causes of waste.

Causes of waste	[1]	[2]	[3]	[4]	[5]	[6]	[7]	[8]
**Design**								
**Frequent design changes**		√		√		√		
**Mistakes and errors in design**		√			√			√
**Workmanship**								
**Using untrained laborers**		√	√					
**Redoing work due to workers’ mistakes**		√				√		√
**Lack of skills**			√	√		√	√	
**Lack of experience**			√	√				√
**Management**								
**Poor site management and supervision**				√		√	√	√
**Inadequate planning and scheduling**							√	√
**Ineffective communication**			√					
**Selecting the lowest bidder contractor/subcontractor**		√						
**Making-do/waiting for prerequisite work**			√					
**Material**								
**Wastage of cement if stored for many days due to moisture**	√							
**Unnecessary material allocations**	√							
**Improper cutting of materials**	√					√		
**Selection and supply of material noncompliance to the specification and quality**	√		√		√	√		
**Improper storage**							√	
**Poor material handling**				√				
**Machinery**								
**Equipment malfunction**							√	
**Others**								
**Rework/repair of defective work**			√					
**Inaccessibility for maintenance work**					√			

[1] Musarat et al. (2023); [2] Mahamid (2022); [3] Igwe et al. (2022); [4] Fitchett and Rambuwani (2022); [5] Islam et al. (2021); [6] Sweis et al. (2021); [7] Arshad et al. (2018); [8] Nagapan et al. (2012).

Inadequate planning and scheduling often manifests in the form of ineffective communication and inaccurate procurement processes. These deficiencies can lead to miscoordination and delays, resulting in unnecessary waste. For example, when planning and scheduling are inadequate, materials may be ordered too early or too late, leading to improper storage or the need to accelerate work, both of which can cause damage or deterioration of materials. Such scenarios increase the likelihood of defects occurring, which subsequently require rework or replacement, thus contributing to C&D waste. The direct link between inadequate planning and the occurrence of defects further underscores the importance of addressing this factor to minimize waste.

Frequent design changes and modifications during construction (Sweis et al., 2021) is another top cause of waste. These changes often necessitate rework, which consumes additional materials and generates considerable waste. For instance, when a design change is made during construction, it can lead to the demolition of already completed work, resulting in the wastage of both labor and materials. Furthermore, defects arising from initial design errors or omissions can exacerbate this issue, as they may require significant alterations or corrections during the construction phase. The interplay between design changes and construction defects thus amplifies the impact on waste generation, highlighting the critical role of accurate and comprehensive design in waste reduction.

### Causes of rework

Studies identifying the causes of rework have the second-highest number of publications. Table 2 illustrates the identified causes and the nature of the rework. Out of the causes affecting rework in construction sites, Mahamid (2022) ranked errors and omissions as the predominant cause. According to Yap and Tan (2021), inadequate quality management, improper planning, communication deficiencies, design alterations, and ineffective subcontractor management stand out as the primary causes of rework in Malaysia. These causes often lead to construction defects that require rework, significantly increasing construction waste. Rework, particularly when driven by defects such as errors in design or noncompliance with specifications, involves the removal or correction of faulty work, which generates waste not only in terms of discarded materials but also in the additional resources needed for corrective measures. This cyclical process of rework due to defects underscores the high impact of defects on construction waste, making the identification and rectification of defects crucial in reducing waste.

**Table 2.** Causes of rework.

Causes of rework	[1]	[2]	[3]	[4]	[5]	[6]
**Design**						
**Errors and omissions**	√				√	√
**Nonconformance with specification**	√					
**Scope changes**	√			√		
**Item not provided in bill of quantities**		√				
**Negligence of design detail by design consultant**		√				
**Workmanship**						
**Lack of labor skills**	√					
**Management**						
**Inadequate supervision**	√	√	√			
**Mismanagement**		√				
**Poor coordination between the design team and site personnel**		√		√	√	
**Poor quality management**				√		
**Poor subcontractor management**				√		
**Material**						
**Incorrect or defective material usage**			√			

[1] Mahamid (2022); [2] Singh et al. (2022); [3] Mostofi et al. (2022); [4] Yap and Tan (2021); [5] Josephson et al. (2002); [6] Love and Li (2000).

Enshassil et al. (2009) analyzed 92 factors contributing to material waste and identified rework resulting from noncompliance with drawings and specifications, errors by workers, inefficient material cutting, procurement of noncompliant materials, and improper storage causing damage or deterioration as the five most significant sources of material waste in construction. Defects arising from poor workmanship or inadequate quality management further exacerbate waste, as these flaws often necessitate repeated cycles of rework. This cyclical nature of rework due to defects significantly impacts construction waste, highlighting the importance of addressing and rectifying defects to mitigate waste.

### Cost of rework

Research on the cost of rework was the next most significant area of research, with 12 records published on this topic during the selected period. The early estimation of defect waste costs provides several benefits for professionals in the building construction sector to ensure the construction budget is ready to deal with defect risk. The argument that the cost of poor quality often exceeds the investment required to manage quality (Johnsson & Meiling, 2009) highlights the importance of understanding these costs. Addressing defects effectively can reduce overall waste generation and improve cost management.

Hwang et al. (2009) analyzed data from 359 construction projects in the Construction Industry Institute database and found that design errors and omissions, along with owner changes, significantly increased construction costs compared with other sources of rework. Josephson et al. (2002) identified that design-related issues, contributing 26% of the rework costs, were the largest factor, with product management issues contributing 25% and erroneous workmanship adding 13%. These findings illustrate how defects and their associated rework costs can affect overall construction expenses and waste generation, providing insights into how to mitigate defects and reduce waste.

Case studies by Love and Li (2000) and Love and Sohal (2003) showed that the cost of rework in the two case projects was 3.15% and 2.40% of the project contract value, respectively, with design changes and errors being major contributors. The studies revealed that defects accounted for 1.5% of rework costs in a residential apartment and 40% in an industrial warehouse. This variation underscores the need to explore the relationship between defects and waste generation. Understanding how different types of defects contribute to rework costs can help in developing effective management practices to reduce both defects and associated waste.

Simpeh et al. (2012) found that direct and indirect rework costs averaged 2.93% and 2.20% of the contract value, respectively, emphasizing the significant impact of rework on project cost overruns. Oke and Ugoje (2013) identified that the highest rework costs were associated with substructures (9.4% of the total average project cost), whereas roof finishes incurred the least. These findings demonstrate how specific aspects of construction are more susceptible to defects and rework costs, contributing to waste generation. Understanding these patterns helps in targeting areas where improved quality management could significantly reduce both costs and waste.

Mahpour (2023) explored the use of machine learning to predict maintenance costs, suggesting that improved cost estimation accuracy can reduce overestimation and resource wastage. Mills et al. (2009) reported that rework costs averaged about 4% of the contract price, whereas Taggart et al. (2014) found that contractors typically allocate 1%–1.25% for snagging, with significant costs associated with defects in various building items. These studies highlight the financial impact of defects and the potential benefits of enhanced quality management and waste reduction strategies. Integrating these insights into construction practices and policies can contribute to minimizing waste and improving sustainability.

### Managing waste from defects

London et al. (2021) proposed a model based on Atkinson's framework (Atkinson, 1999) to manage defects that could lead to waste by linking defects, technology, and quality through complex pathways. This model provides valuable insights into how advanced frameworks can enhance defect management and reduce waste, contributing to more integrated waste management strategies.

Shooshtarian et al. (2023a) introduced a simpler model for managing building defects in residential projects, validated by Australian experts. This model emphasizes the importance of stakeholder collaboration to minimize defects and their resulting waste. It demonstrates how coordinated efforts can effectively manage defects and reduce waste, supporting better quality management practices. Singh et al. (2022) developed a predictive model for identifying potential rework scenarios during the preconstruction and construction phases. This approach helps address defects before they lead to significant waste, improving construction efficiency and reducing overall waste generation. Sharma et al. (2022) argued that strategic building maintenance extends the service life of buildings and delays demolition, thereby postponing the creation of waste from defects. This perspective underscores the importance of proactive maintenance strategies in reducing waste and enhancing sustainability.

Additionally, several studies propose the use of new technologies and tools to minimize the causes of waste, inspect building components, and manage materials effectively before they deteriorate (Atherinis et al., 2018; London et al., 2021; Won et al., 2016). Won et al. (2016). Won et al. (2016) found that BIM can significantly reduce waste by validating designs, detecting clashes, and reviewing design errors. Such technological innovations are crucial for enhancing defect management and reducing overall construction waste.

### Quantifying waste from construction defects

Research specifically addressing the quantity of waste resulting from defects is notably scarce, highlighting a significant gap in the literature. Pan and Thomas (2015) observed that the amount of defect-related waste varies depending on the building type rather than the gross floor area, suggesting that different types of buildings may produce varying volumes of defect waste. Similarly, Hauashdh et al. (2022) noted that the use of inappropriate or low-quality materials contributes significantly to the generation of maintenance waste, further underscoring the need to address material choices to manage defect waste effectively.

In a study by Sun et al. (2020), approximately 106 tons of defect-related waste were reported in Shenzhen, China, mainly during the decoration and maintenance phases. This waste included materials such as brick, concrete, metals, timber, glass, gypsum, tile, and paint, illustrating the diverse nature of waste from defects and the challenge of quantifying it accurately. Although several methods for general C&D waste quantification are available, such as those summarized in Table 3, there is a notable lack of specific methodologies for defect-related waste. These methods, which estimate waste in terms of weight and volume, could potentially be adapted to quantify defect waste more precisely. Addressing this gap in research is crucial for developing more effective strategies to manage and minimize defect-related waste.

**Table 3.** Summary of selected studies that proposed construction and demolition (C&D) waste quantification methods.

Study aim	Findings	Reference
**Establish C&D waste ratio via analysis of 18 building sites in Brazil**	The results showed an average C&D waste generation rate of 0.151 m^3^/ m^2^	Teixeira et al. (2020)
**Estimate C&D waste in building energy efficiency retrofitting works of the vertical envelope**	Study found that C&D waste generated lies between 2.46 and 65.24 kg/m^2^ and 0.012–0.008 m^3^/m^2^	Sáez et al. (2018)
**Develop a formula for C&D waste estimation its volume and weight considering two variables (number of dwellings and total floor area individually) in housing projects**	The results obtained with the model show a mean deviation of around 0.75% in weight and 10.28% in volume, as opposed to the amount of C&D waste generated in other projects	Sáez et al. (2015)
**Generate a selective classification and quantification model of C&D waste based on the material resources consumed in the construction of residential buildings**	The results show that the ratio of waste to material resources consumed in the case studies is 3.6%	Mercader-Moyano and Ramírez-de-Arellano-Agudo (2013)
**Develop a model to quantify construction waste in projects according to the European waste list**	The results show that the ratios of C&D waste generation for three types of waste: remains, packaging, and zearth/soil are 0.057, 0.082, and 0.281 m^3^/m^2^ for earth	Llatas (2011)
**Develop a procedure guiding on-site waste sorting and weighing in four ongoing construction projects in Shenzhen city**	The results revealed that the total waste ratio ranged from 3.275 to 8.791 kg/m^2^	Lu et al. (2011)
**Establish a model for the quantification of C&D waste based on the project budget.**	The results revealed that the waste generation ratios are 0.031 m^3^/m^2^ for new construction works and 1.268 m^3^/m^2^ for demolition works.	Solís-Guzmán et al. (2009)

By improving our understanding and quantification of defect-related waste, this study aims to contribute to more accurate waste management practices and enhance the sustainability of construction projects. The development and application of precise quantification methods will help in identifying patterns, trends, and areas for improvement in managing waste associated with defects.

## Discussion

### Discussion of key findings

The findings reveal a growing number of studies addressing construction defects, indicating an increasing interest in this area of research. This growth aligns with the trends noted by Gurmu et al. (2022), who also observed a heightened focus on defects within the building construction sector. This study adds to this body of literature by highlighting that the increasing attention is not merely academic; it reflects the industry's recognition of the significant impact that defects have on overall construction quality and waste generation. Furthermore, the introduction of new technologies, such as BIM, augmented reality, virtual reality, and AI, as tools to minimize defects and associated waste, marks a promising development in the field. This is supported by Desai et al. (2023), who also emphasize the role of these technologies in defect reduction. However, the findings of this study suggest that although these technologies are being increasingly adopted, their full potential in minimizing defect waste is yet to be realized, requiring more widespread implementation and integration into standard construction practices.

Regarding geographical trends, the study found that Malaysia has the greatest number of publications in this field. This result is consistent with Saadi and Ismail (2015), who identified Malaysia's rapid economic growth and urbanization as drivers for increased construction activities and, consequently, more research in this area. However, whereas Saadi and Ismail (2015) attribute this primarily to regulatory frameworks like the Solid Waste and Public Cleansing Management Act 2007, this study’s findings suggest that Malaysia’s proactive stance on sustainable construction practices and waste management has also contributed to the heightened research interest.

The primary causes of defect waste identified in this study—poor workmanship, inadequate planning, and design errors or changes—are consistent with the findings of London et al. (2021) and Taggart et al. (2014), who also highlight these factors as significant contributors to construction waste. However, although previous studies focus on these issues as general contributors to waste, this research specifically connects these factors to defects, emphasizing that defects arising from these causes can be reasonably managed through effective collaboration between various stakeholders. This finding is particularly important because it suggests that addressing these root causes through improved communication, planning, and coordination among construction teams could significantly reduce the occurrence of defects, and consequently, the associated waste. This perspective adds a layer of nuance to the existing literature, which often treats these causes in isolation rather than as interconnected contributors to defect waste.

The cost of waste is expected to become an increasingly important issue in construction costs, as also noted by Moschen-Schimek et al. (2023). Their research on waste disposal costs aligns with this study’s findings that such costs are rising, driven by policies aimed at encouraging a circular economy in the construction sector. However, this study goes farther by linking these rising costs directly to the issue of defects. The surges in building insurance premiums and the complexities of filing claims, as discussed by Ali et al. (2020), are exacerbated when construction defects are involved. The findings of this research suggest that defects not only contribute to increased waste disposal costs but also complicate the insurance claims process, adding financial and procedural burdens to the parties involved. This connection between defects, waste costs, and insurance challenges adds a new dimension to the existing literature, emphasizing the multifaceted impact of defects on construction economics.

In terms of waste due to defect quantification, the findings of this research highlight the need for a comprehensive procedure that considers all internal and external factors influencing waste quantity. This aligns with the critiques of existing methods by Sáez et al. (2015) and Mercader-Moyano and Ramírez-de-Arellano-Agudo (2013), who point out the complexity and inaccuracies in current waste estimation approaches. However, this study diverges by focusing specifically on the challenges posed by defects in waste quantification. It is argued that the existing methods are particularly inadequate for accurately estimating waste emanating from defects due to their failure to account for the unique characteristics of defects, such as variability in defect types and their impact on overall waste generation. This finding underscores the necessity for developing specialized estimation methods tailored to the unique nature of waste from defects, a gap that has been insufficiently addressed in the current literature.

Finally, the research underscores the importance of developing a system to avoid, detect, and rectify construction defects early. Previous studies by Shooshtarian et al. (2023b) and Rotimi et al. (2015) highlight the need for user-friendly systems to enhance defect management. The findings of this research corroborate this view but also emphasize that the effectiveness of such systems is contingent on their ability to be integrated seamlessly into existing construction workflows. By ensuring that these systems are not only user-friendly but also adaptable to various project scales and complexities, the industry can achieve a significant reduction in defect-related waste. This focus on integration and adaptability adds to the current understanding of defect management systems, suggesting that their design should prioritize ease of use alongside functional effectiveness.

### Future research directions

This research sheds light on the existing focus on waste generated due to defects in the construction industry, highlighting several critical gaps and future needs. Despite a substantial number of studies addressing defects, rework, and waste, the interplay among these factors has received comparatively less attention. This research suggests that understanding the process and causes of defect-rework-waste generation remains underexplored. Future research should therefore focus on elucidating these interrelationships to develop a comprehensive understanding of how defects contribute to rework and waste and vice versa.

Additionally, the analysis indicates a trend in current research towards minimizing construction waste, identifying causes of construction defects, quantifying the cost of rework, and understanding the causes of both rework and waste. However, there is a notable lack of emphasis on several critical areas. Specifically, quantifying the cost of defects, developing strategies to minimize defects and rework, and exploring the relationships between defects, rework, and waste have been relatively neglected. Future research should address these gaps in four ways. The first is by quantifying the cost of defects. More detailed studies are needed to assess the financial impact of defects on construction projects. This includes developing methodologies to accurately quantify defect-related costs and integrating these findings into cost management practices. The second is by developing strategies to minimize defects and rework: Research should focus on identifying and implementing effective strategies for reducing defects and rework. This includes exploring technological solutions, process improvements, and best practices that can be applied across different types of construction projects. The third is by exploring relationships between defect, rework, and waste. Future studies should investigate the interconnectedness of defects, rework, and waste to develop a holistic approach to defect management. This could involve developing models or frameworks that illustrate how these factors interact and influence each other. Finally, the fourth is by developing integrated management systems. There is a need for research into integrated systems that combine defect management, rework reduction, and waste minimization. Such systems should be user-friendly and adaptable to various project scales and complexities, facilitating their adoption by industry professionals. By addressing these deficiencies, future research can contribute to more effective defect-rework-waste management practices and enhance overall construction project efficiency. The findings of this study underscore the importance of these research directions, providing a foundation for future investigations and developments in the field.

### Implications and validity of the study

The findings of this research provide valuable insights into the management of construction defects and waste, with implications for the broader building construction sector. The observed increase in research and technological advancements reflects a sector-wide trend towards addressing these critical issues. By highlighting key causes of waste such as poor workmanship, inadequate planning, and design errors, this study underscores challenges that are prevalent across various construction projects from residential to commercial and infrastructure.

The practical importance of these findings is evidenced by their alignment with industry needs for effective defect and waste management strategies. Recommendations derived from this research, such as improved stakeholder collaboration and the adoption of new technologies, are directly applicable to enhancing construction practices. These recommendations are further supported by existing literature and case studies demonstrating the successful implementation of similar strategies in the industry. Furthermore, the research highlights actionable steps for industry professionals. The connection between defect management and waste reduction can inform practices and policies, such as those seen in Malaysia's regulatory frameworks, which align with the findings of this study. This suggests that the study’s insights are not only valid but also valuable for guiding industry practices.

To ensure the validity of the research, a robust methodological framework (PRISMA) was used and supported by a comprehensive analysis of relevant literature and data. The alignment of these findings with previous research underscores their reliability and applicability to real-world scenarios. Future research should focus on further validating these findings through empirical studies in different construction contexts and exploring the effectiveness of proposed strategies in real-world settings. This will enhance the generalizability of the results and provide a more comprehensive understanding of their impact on the building construction sector.

## Conclusion

This study highlighted the critical role of construction defects as significant contributors to C&D waste, drawing attention to the intricate relationship between defects, rework, and waste in the construction industry. The findings reveal a growing body of research focused on these issues, with a marked increase in studies since 2009. This trend highlights the industry's recognition of defects as a major challenge to construction quality and waste management. The study’s analysis identifies poor workmanship, inadequate planning, and design errors or changes as primary causes of defect-related waste. These issues are not only prevalent but can be mitigated through improved stakeholder collaboration, effective planning, and the adoption of emerging technologies like BIM, augmented reality, virtual reality, and AI. These technologies, although promising, require broader adoption and integration into standard practices to fully realize their potential in reducing defect-related waste. The study also identifies the growing financial implications of construction defects, particularly in the context of rising waste disposal costs and increased complexities in building insurance claims. These economic pressures further underscore the importance of addressing defects to reduce waste and associated costs.

However, the research is not without limitations. The reliance on data from Scopus and Web of Science (WoS) may have restricted the scope of the study, potentially overlooking relevant research outside these databases. Additionally, the specific keywords used in the analysis might not encompass all pertinent studies, suggesting a need for broader keyword selection in future research. Despite these limitations, the study makes significant contributions to both theory and practice. It provides a detailed review of literature on defect-related waste, offering a foundation for future research that could explore new methodologies for quantifying defect-related waste and strategies for its reduction. The identification of 12 subthemes for further investigation presents a roadmap for advancing research in this domain. Practically, the findings offer actionable insights for industry professionals, emphasizing the need for improved defect management strategies and the integration of advanced technologies to minimize waste. From a policy perspective, the study’s results can inform the development of regulations aimed at reducing postconstruction waste, aligning with broader goals of sustainability and the circular economy.

## Data Availability

All journals used for review are listed in the references.
